# The importance of electrode interfaces and interphases for rechargeable metal batteries

**DOI:** 10.1038/s41467-021-26481-8

**Published:** 2021-10-29

**Authors:** Jelena Popovic

**Affiliations:** Max Planck Institute for Solid State Research, Heisenbergstr. 1, 70569 Stuttgart, Germany

**Keywords:** Materials chemistry, Batteries, Energy, Batteries, Electrochemistry

## Abstract

Rechargeable metal batteries are one of the most investigated electrochemical energy storage system at academic and industrial level because of their possibility to store higher energy compared to their counterparts employing carbon as an anode material. However, to produce reliable and durable metal batteries, it is of paramount importance to understand and circumvent (or ultimately overcome) the issues associated with the chemically reactive, ionically blocking and mechanically unstable interfaces and interphases of the metal electrode. Here, recent progress and the future perspective of this field are discussed from a physicochemical perspective while, at the same time, fundamentally relevant  questions are raised.

## Abstract (web summary)

Metal electrode interfaces and interphases are critical for the development of future high-energy metal batteries. Here, Dr Jelena Popovic-Neuber discusses the state of the art, issues and strategies to improve the stability of metal electrodes toward practical battery systems.

Battery technologies play a key role in the transformation of the current energy consumption from fossil fuel to renewable alternatives. In the last decades, rechargeable Li-based batteries have become ubiquitous in everyday life. However, further increase of their energy density relies on the possibility to use alkali (e.g., Li, Na, K) and alkaline earth metals (e.g., Mg, Ca) as anodes in alternative technologies such as metal-sulfur and metal-air battery cells. Interestingly, one of the first commercial battery cells for laptops and cell phones (e.g., Molicel) utilized lithium metal as an anode material^1^. Molicel cells and similar technologies had to be withdrawn from the global market at the end of the 80s because they were prone to catch fire under standard operation due to internal-short circuits and thermal runaway^1^. After more than 30 years from this commercial failure, metal batteries are back in the research laboratories of industry and academia. The main issues which still remain to be addressed can be summarized as follows:

1. Presence of non-passivating (i.e., porous, chemically reactive) or ion-blocking interphase (i.e., electrolyte-derived solid electrolyte interphase, SEI) on the metal electrode.
2. Electrodeposition (i.e., the reduction of the metal ion sourced from the electrolyte into zero valence state on the metal electrode surface upon current application) issues associated with the growth of metal in different non-flat forms (e.g., dendrites, mossy deposits, balls and whiskers^2^) leading to short circuits, increased cell resistances, and electronically disconnected parts of the metal electrode (i.e., formation of “dead” Li).
3. Large volumetric and surface area changes of the metal electrode upon cycling.

The origins of metal cell failures lie in the physicochemical properties of the metals, including their high reactivity in the pristine state, as well as differences in the surface and bulk ion diffusion kinetics. Furthermore, both the nature of electrodeposition and underlying electrode/liquid electrolyte interface are influenced by the molecular structure of electrolyte, which is largely a function of ionic radii and corresponding charge densities. The current understanding is that, in all cases, SEI control is the key factor for enabling metal batteries. Figure 1 shows some of the above-mentioned issues and potential solutions, which are discussed in detail below.

**Fig. 1:**
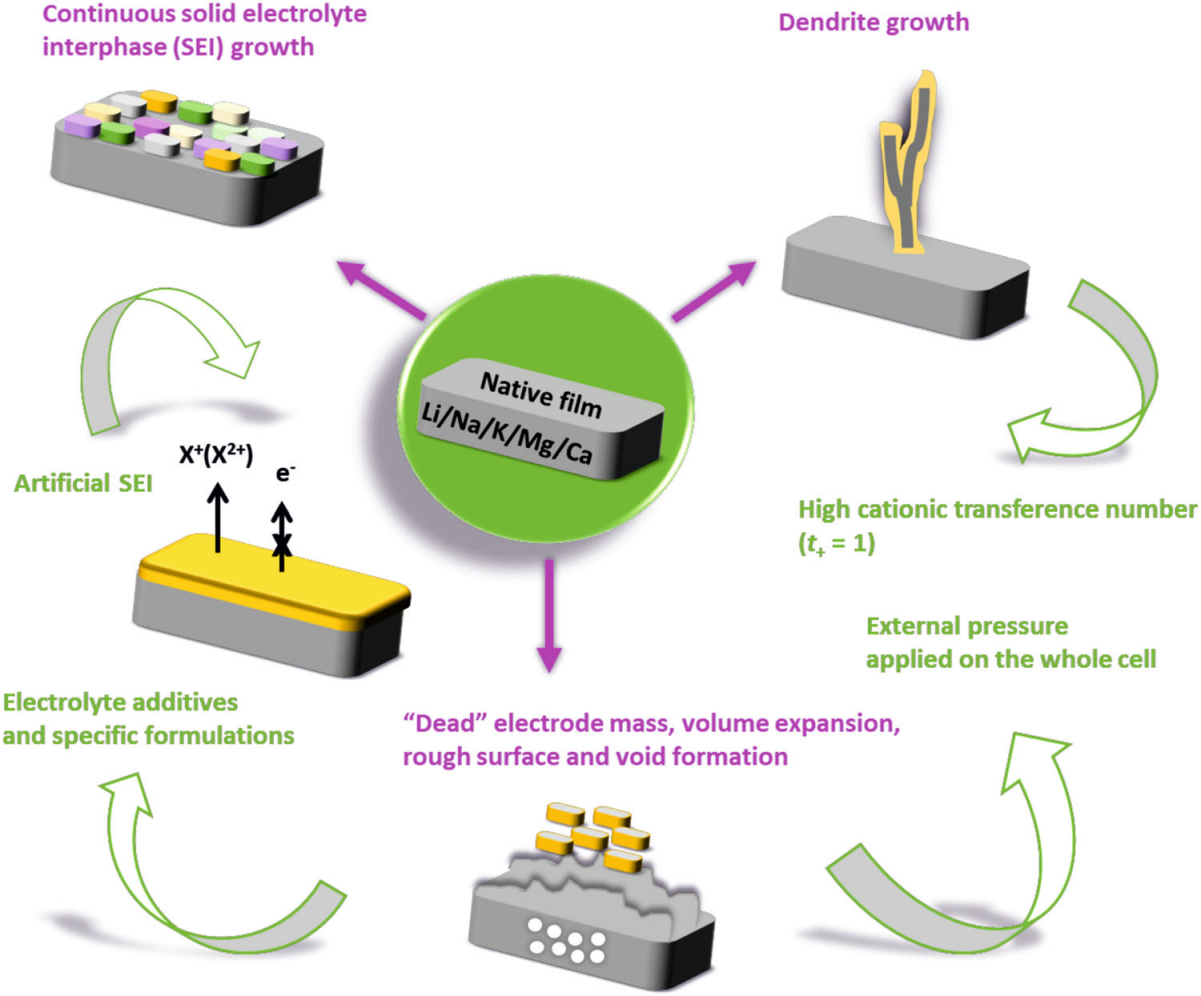
Issues (marked in magenta) and circumvention/overcoming strategies (marked in green) in metal anode cells. In the upper left part, the different colors represent the heterogeneous chemical composition of the solid electrolyte interphase (SEI). The sketch of SEI is oversimplified since the grain size and shape vary considerably, and grains grow on top of each other in a disordered way. In the rest of the figure, SEI is represented in orange. The use of inorganic and purely ionically conductive artificial SEIs circumvent the continuous SEI growth and limit the formation of “dead” (i.e., electronically disconnected) active material. Electrolytes with high cationic transference number show beneficial influence on reducing the formation of dendrites. Application of external pressure on the whole cell and use of electrolyte additives or specific formulations positively help tackling the issues associated with volume expansion, rough electrode surface, and void formation. Electrolyte additives and specific formulations can also positively affect the formation of mechanically and electrochemically stable SEI.

## Solid electrolyte interphase

Alkali and alkaline earth metals form a thin native surface film consisting of their oxide, hydroxide, and carbonate compounds if residual hydrocarbons, CO_2_ or water are available, even if the metals are of high purity, stored under Ar-atmosphere, and freshly cut before usage. According to the Pilling–Bedworth ratios (i.e., the ratio of the volume of the elementary cell of a metal oxide to the volume of the elementary cell of the metal from which the oxide is formed) and experimental evidence (mostly available for Li)^3^, formation of several nanometer-thick, chemically inhomogeneous, and porous native films occurs. Therefore, the liquid and soft solid-state electrolyte can easily infiltrate these films. It remains unclear if and under which circumstances electron and ion transport is possible through the native films. In the worst-case scenario, it can be speculated that some electrode areas become adversely isolated, even before cell assembly.

Besides the native surface film, metal electrodes generate an SEI which is a hybrid (organic/inorganic) passivation film formed on the electrode surface when in contact with salts, solvents, additives, and impurities of liquid electrolyte during chemical (e.g., corrosion during calendar ageing) or electrochemical (e.g., current or voltage perturbations) conditions. SEI on alkali and alkali earth metals can be several hundreds of nanometers thick and is commonly represented by a mosaic structural model in which different materials (e.g., carbonates, oxides, fluorides, fluorophosphates, polyolefins, semicarbonates, alkoxides, polymers, and others) are treated as hetero-poly-microphases, with possible porosity on the surface towards the electrolyte^4,5^. Ideally, SEI should grow rapidly and provide full coverage to avoid continuous irreversible electrolyte consumption. Upon formation, the SEI should behave as a cationic conductor, encompassing both negligible electronic conductivity and good mechanical properties such as high deformation energy. Although the SEI has been thoroughly studied in the past, experimental limitations related to the spatial averaging of collected spectroscopic and microscopic data and measurements sensitivity leave various questions open: Is the SEI crystalline or partly amorphous? Are the inorganic components of the SEI highly defective, or can we rely on their known bulk conductivity? How does the molecular composition and morphology of the SEI affect the ion transport? How chemically and morphologically dynamic is the SEI upon cell cycling? Is the SEI porous or a dense layer? Is it possible to develop a general SEI model dealing with growth, transport, and ageing, or specific chemistries are decisive?

In early research on the stabilization of the alkali metals SEI, non-aqueous carbonate and ether-based electrolyte components were varied by either rational design or trial-and-error approaches with the final idea of producing predominantly crystalline, inorganic SEIs (e.g., fluoride, nitride, iodide, and carbonate). It was demonstrated that higher salt concentrations are beneficial, while electrolyte additives such as fluoroethylene carbonate and CO_2_ in fluorinated solvents improve Coulombic efficiency (i.e., the ratio of discharge to charge capacity in the same cycle)^6–9^. In these cases, since the additives also get slowly reduced into SEI, their concentration decreases with time, making electrode degradation still possible and heavily kinetically controlled. As a potential improvement, SEIs prepared directly on alkali metals from liquid and gaseous phases (also called artificial SEIs) before the cell assembly are suggested^10^. However, unlike the SEIs prepared upon contact with liquid electrolyte after cell assembly, artificial SEIs do not possess necessary self-healing properties when fractured. It is also unlikely that a wide variety of artificial SEIs can be produced as dense and chemically/mechanically homogeneous layers.

Compared to Li, SEIs formed on Na, K, Mg, and Ca metal electrodes recently received increased attention from the scientific research community. In Mg/Ca metal batteries the issue lies mainly in the inability to deposit the metal on the electrode surface^11^. For example, it is reported that SEI formed on Na metal is partially soluble in alkyl carbonate electrolytes while, in ether-based electrolytes, the SEI on Na metal shows unexpectedly low initial resistances and transport instabilities after long-term ageing. Also, SEIs formed on K metal electrodes are reported as mechanically unstable, while SEIs on Mg metal electrodes formed during contact with glyme-based electrolytes are possibly composite in nature (comprising liquid and solid phases)^12–14^.

## Dendritic growth and electrodeposition problems

The native surfaces of metal electrodes contain inherent dislocations, ridges, cracks, and striations which causing energetically favorable specific electrodeposition areas^15,16^. Dendrites or protrusions (i.e., penetrating electrodeposition products) additionally occur due to the space charge zone formation under current load (above c. 1 mA cm^−2^), a consequence of concentration polarization of the liquid electrolyte. Following the theoretical Sand’s time (i.e., time at which metallic deposition products start to grow) and experimental measurements, it was found that the increase of dendrites growth is favored in high applied currents (e.g., up to 9 mA cm^−2^ for Li metal), low salt diffusion coefficients, and in electrolytes with low cationic transference numbers^17,18^. Interestingly, at very high currents (e.g., 15 mA cm^−2^) the internal self-heating may trigger surface diffusion which smoothens the dendrites^18^. The protrusions are also expected to appear due to non-uniform SEI breakdown (e.g., solubility or mechanical instability) and current distribution inhomogeneity. The latter aspect is closely related to the chemical properties of the SEI, as well as with concurrent growth of SEI and dendrites. Also in this case, a number of fundamental questions remain open: Are protrusions always formed on top of the metal electrode, or can plating be possible also on the SEI when free electrons are available? How does the ion transport in the SEI affect the dendrite formation, shape and growth speed? How significant is the influence of the cell pressure on the dendrite shape and propagation? How do the electron and mass transfer resistances as well as electrolyte speciation influence the alkali and alkali metal deposition?

Initial dendrite growth can be observed by *operando* experiments, e.g., using specially designed cells for cryogenic transmission electron microscopy measurements. Under those conditions, which can be significantly different from the ones in practical cells, it has been shown that Li dendrites are single-crystalline nanowires that change growth directions due to SEI formation^19^. The kinking and buckling of dendrites leads to the formation of “dead” Li, thus columnar growth with low tortuosity should be aimed at. The importance of alkali hydride species sometimes observed on the dendrite tips is still under intense discussion within the research community. Unfortunately, even in well-optimized liquid electrolytes, the deposition of lithium remains porous on the micrometer scale. Upon stripping at higher currents (in liquid electrolytes already around 1 mA cm^−2^ for Li), alkali metal vacancies coalesce, creating voids above which the SEI collapses^20^.

In most of the non-aqueous liquid electrolytes, self-electrodeposition of Mg and Ca is unattainable due to poor salt solubility and formation of ionically blocking SEI. In particular, in Mg metal electrodes, the halide anions are important electrolyte components that serve as ligands, improving salt dissolution corroding the blocking SEI layer or through adsorption on the electrode surface^21^. Ca self-electrodeposition is possible at room temperature in specific electrolyte chemistries with boron-based salts and when fluoride and halides are dominating the SEI chemistry^22,23^.

## All-solid-state metal cells

The issues in all-solid-state metal cells (where the electrolyte is present as a solid-state material, either organic or inorganic) are mainly related to the chemo-mechanical properties of the solid-state electrolytes such as cracking, poor contacts inducing current constriction, dendrite growth in the proximity of imperfections (e.g., voids, grain boundaries, impurities and local gradients of the cationic transference number), and kinetically unstable interfaces allowing for the growth of ionically blocking, electronically conductive or chemically reactive phases. Flat and atomically intact metal surfaces, use of alloys as negative electrode active materials, heat treatment and high stack pressure (300–1000 MPa) application during cell preparation, as well as employment of sulfide-based electrolytes linked with more chemically stable argyrodite-based interfaces, are proposed to address the all-solid-state metal cell issues^24,25^. Several theoretical models are currently being developed to estimate critical current density upon stripping of the metal electrode above which voids and dendrites form^26,27^. Viscoelastic creep plays an essential role in this case. In this regard, Na is expected to be a slightly more promising electrode material compared to Li^28^. In the case of Mg and Ca metal electrodes, ionic transport in the solid-state electrolyte is the rate-determining step and needs to be considerably improved. Sluggish mobility of bivalent cations may be addressed by high volume per anion or metastable structures^29^.

Finally, the quest for finding an organic polymer matrix providing sufficient salt solubility, decent room-temperature conductivity, high cationic transference number, and stable interfaces is still a vigorous area of research. Hybrid organic polymer-inorganic solid electrolytes with improved mechanical and interfacial properties are also heavily discussed as potential solutions as they offer good wetting and additional inorganic or interfacial conduction pathways^30^.

## Next-generation characterization techniques and modeling

To answer the open questions mentioned above, a deeper understanding of how physicochemical characterization techniques can be applied at high spatial and possibly atomic resolution is required. In particular, the development of innovative *operando* measurements investigating local ion transport and the structure of interfaces is of utmost importance. *Operando* techniques based on synchrotron X-ray and neutron diffraction coupled with computed tomography and profiling measurements, as well magnetic response-based analytical methods, are in their early stage of development and highly promising to help researchers better understand both electrode/electrolyte interfaces and interphases. Suitable models for charge transfer, simultaneous metal electrodeposition and SEI growth on metal electrodes still need to be developed. In the future, the issues associated with the ionic concentration gradients at abrupt liquid/solid and solid/solid interfaces (i.e., the electrochemical double layer and space charge zones that govern the transport at the phase boundaries) should also be addressed both theoretically and experimentally. These aspects are particularly important in complex battery systems such as metal–sulfur, metal–air, and all-solid-state cells where detrimentally reactive (so-called solid–liquid electrolyte interface) and highly metal-ion conductive space charge zones are present^31,32^.

To understand the dissimilarities between the SEIs and electrodeposition growth mechanisms for different alkali and alkaline metal electrodes, in-depth studies which combine experimental data (e.g., electrochemical impedance and X-ray photoelectron spectroscopy measurements) and modeling efforts (e.g., spectral fitting, equivalent circuit and transmission line models) are required. In the not-so-distant future, this might be possible with fully automated robotic data collection and new computational infrastructures. Nevertheless, supervision of expert scientists, engineers, and reseachers on the fundamental science and engineering level will remain essential.

## Towards practical systems

The lack of consensus in choosing a standard electrochemical cell set-up to investigate the electrochemical energy storage behavior of electrodes at the lab scale level remains one of the major issues in the battery research community. Although it was already pointed out that using lab-scale cells with flooded liquid electrolyte conditions and thick metal electrodes (which are far away from practical large-scale cell systems) allow for observation of apparently good cell performances (even when “dead” electrode active materials are formed), this type of set-up is still considered as the experimental standard in the industry and academic research laboratories^33^. Recently, anode-free cells (i.e., where the negative electrode is in situ formed by electrodeposition of lithium, sourced from the positive electrode, on an electronically conductive matrix) have been considered for offering energy density advantage compared to conventional metal-ion cells^34,35^. In anode-free cells, alkali or alkaline metal inventory loss during electrodeposition or SEI formation is more easily detectable.

Taking into account all these considerations, it is foreseeable that, in the following decades, research activities focusing on rechargeable metal batteries will provide fundamental knowledge on chemical electrode/electrolyte interface and interphase control, as well as many new ideas to develop more sustainable and environmentally friendly battery systems.
